# Intermittent reproduction, mortality patterns and lifetime breeding frequency of females in a population of the adder (*Vipera beru*s)

**DOI:** 10.7717/peerj.6912

**Published:** 2019-05-09

**Authors:** Dirk Bauwens, Katja Claus

**Affiliations:** Department of Biology, Laboratory of Functional Morphology, University of Antwerp, Wilrijk, Belgium

**Keywords:** Capture-recapture, Mortality, Reproduction, Breeding frequency, Snake, Maturity, Costs

## Abstract

Female adders (*Vipera berus*) are “capital breeders” that exhibit delayed maturity and intermittent reproductive frequency. We studied the attainment of sexual maturity, the initiation of annual breeding in mature females, the energy and mortality costs associated with breeding, the length of the reproductive cycle and female lifetime reproductive frequency. We use longitudinal data obtained during an 18-year (2000–2017) mark–recapture study in a large population of adders in northern Belgium. A minority (15%) of the females gave birth to their first litter when they attained the actual age of 3 years, upon surpassing a minimum snout-vent-length of 38 cm. However, most females reproduced for the first time when they were 4 years or older. In mature females, breeding in a given year depends to a large extent on their body condition at the onset of the active season, indicating that a threshold level of energy reserves is necessary to start a reproductive cycle. During breeding years females stop growing, lose about one-third of their initial body mass and are very emaciated after parturition. The decrease in relative body mass was most notable initially in the largest and fattiest females. During the non-breeding years, females forage intensely to rebuild their fat reserves; their abilities to do so will strongly affect whether and when they engage in subsequent reproductive bouts. We used a multistate mark–recapture analytical method to simultaneously estimate the capture and survival rates of breeding and non-breeding females, a necessary procedure to obtain accurate estimates of survival probabilities. The analysis indicated much higher capture rates during the breeding years, but did not reveal a substantial effect of reproductive state on annual survival rates. Although some females were observed to breed in successive years, the reproductive cycle was most often biennial or triennial. However, most females (ca. 70%) that attained sexual maturity reproduced only once during their lifetime, while a minority of the females (ca. 5%) were observed to breed on 3–5 annual occasions. On average, females produced only ca. 1.3 litters during their reproductive lifetime. The short reproductive lifetime is a consequence not only of mortality directly related to the reproductive activities, but also of mortality associated with recovering from the weakened post-parturient body condition during the long intervals (1–2 years) between reproductive bouts.

## Introduction

A fundamental aspect of life-history variation concerns the temporal separation between the acquisition of energy and its investment in reproduction. Some species use freshly acquired energy to fuel reproduction (“income breeders”), whereas other species acquire and store energy over long periods prior to its expenditure in reproduction (“capital breeders”, Drent & Daan, 1980). This “capital breeding” strategy is particularly common in ectotherms, especially in long-lived lizards and snakes (Bonnet, Bradshaw & Shine, 1998). Many of these species require long periods to grow to mature size and hence exhibit delayed maturity (Dunham, Miles & Reznick, 1988). In addition, upon attaining maturity, these species typically invest large amounts of stored lipids in their first reproduction, resulting in a depletion of the body reserves (Madsen & Shine, 1993; Bonnet et al., 2002; Brown, 2016). Subsequently, it may take one or more non-breeding seasons to replenish energy reserves to levels required for the induction of the next reproductive episode. This may result in patterns of intermittent reproduction, whereby reproductively mature individuals may be incapable of breeding in successive years (Bonnet et al., 2002; Brown, Kéry & Hines, 2007; Schuett, Repp & Hoss, 2011; Baron et al., 2013; Brown, 2016). This is expected to occur when resource availability strongly constrains reproduction and when the cost of breeding includes a substantial “overhead” component that is partly independent of fecundity (Bull & Shine, 1979).

Understanding the factors that have moulded a particular suite of life history traits in a population or species requires a detailed knowledge of the nature of the costs associated with reproduction. Costs of reproduction can take several forms. The most common are an increased risk of mortality (“survival costs”) and a trade-off induced by the allocation of energy reserves into present reproduction (“energy costs”) (Williams, 1966; Stearns, 1992). The latter may represent the consequences of a reduced body condition and/or decreased growth rates on the fecundity at future reproductive events. The exact forms of reproductive costs are dependent on how reproduction affects the general condition and behaviour of the organism, affecting its susceptibility to different mortality agents, and on how the amount of energy allocated to current reproduction influences the success of future reproductive episodes (Shine, 1980; Bonnet et al., 2002).

Viviparous snakes, especially *Vipera*-species that inhabit temperate regions with strong seasonality, are excellent organisms in which to study reproductive trade-offs. These species are typical capital breeders and provide no parental care (Madsen & Shine, 1993; Bonnet, Bradshaw & Shine, 1998; Bonnet et al., 2002; Baron et al., 2013). At ovulation, females convert the previously assembled energy stores into yolk, the exclusive source of nutrition for the developing embryos (“lecithotrophy”, (Stewart, 2015)). The high energy investment at vitellogenesis and the substantial metabolic costs of pregnancy result in a depletion of the body reserves by the time of parturition (Nilson, 1981; Madsen & Shine, 1993; Bonnet et al., 2002). Replenishment of the energy reserves may then require one to several years, such that females cannot produce litters every year (Madsen & Shine, 1993; Bonnet, Bradshaw & Shine, 1998; Bonnet et al., 2002; Pleguezuelos et al., 2007; Baron et al., 2013). The concurrent presence in a population of adult females in their reproductive and non-reproductive years offers an opportunity to estimate the costs associated with reproduction. These should be reflected in reduced survival and/or lower growth rates of reproductive versus non-reproductive individuals.

We here report results of a citizen science project conducted continuously over an 18-year period (2000–2017) in a large population of European adders (*Vipera berus*). Numerous studies have addressed various aspects of this species’ life history, demography, behaviour and ecophysiology (e.g., Prestt, 1971; Völkl & Thiesmeier, 2002; Guillon et al., 2014). However, reliable estimates of reproductive variables and breeding frequency are available for only one small, threatened population in southern Sweden (Madsen & Shine, 1992; Madsen & Shine, 1993; Madsen & Shine, 1994). By contrast, our capture–recapture study provided longitudinal data for a large sample of individually marked adult female snakes. This enables quantification of (lifetime) breeding frequency and of body condition, growth rate and survival probabilities in relation to yearly breeding status. Adult female vipers are typically very secretive and have low catchability during their non-breeding years (Madsen & Shine, 1993; Bonnet & Naulleau, 1996; Baron et al., 2013), complicating the estimation of demographic variables. We therefore use likelihood-based statistical methods that provide robust estimates of capture probabilities, of transition rates between reproductive states and of survival probabilities during breeding and non-breeding years (Lebreton et al., 1992; Cooch & White, 2015; Mazerolle, 2015). In other papers, we used this methodology to analyse age-specific annual survival probabilities (Bauwens & Claus, 2018) and seasonal variation in adult survival rates (Bauwens & Claus, 2019a) in our study population.

We address five main questions: (1) Which phenotypic characteristics (age, body size) are associated with the attainment of sexual maturity in female adders? (2) What proximate factors (body size and condition) trigger the initiation of annual breeding in mature females? (3) What are the annual changes in body condition and body size (i.e., growth) during breeding and non-breeding episodes? (4) To what extent are annual survival probabilities influenced by reproduction? (5) What is the length of the reproductive cycle and how frequently do females reproduce during their lifetime?

## Material & Methods

### Study species, study area and data collection

The European adder (*Vipera berus*) is a small, stout-bodied venomous snake that has a huge distribution area covering large parts of Europe and Asia. They typically occur in small, often imperilled populations (10–100 adult individuals) (Madsen, Stille & Shine, 1996; Madsen et al., 2000; Ursenbacher & Monney, 2003; Phelps, 2004). By contrast, in our study area adders are very abundant; total population size is estimated to be in the order of several thousand snakes. A detailed and comprehensive account of the annual cycle in our study population can be found in Bauwens & Claus (2019a) and coincides generally with that observed in other regions (Prestt, 1971; Nilson, 1980; Andrén, 1985; Madsen & Shine, 1993; Madsen et al., 1993; Völkl & Thiesmeier, 2002; Phelps, 2004; Nilson, Andrén & Völkl, 2005).

Data were collected during a long-term citizen science population study (2000–2017) of adders in the “Groot Schietveld” (ca. 1570 ha; N 51°20–22′–E 4°32–37′, province of Antwerp, Belgium). The area is used (since 1893) as a military exercise zone and access is restricted to authorized persons and only when there are no military operations (mainly non-working days and hours). This lowland area (altitude ranges 18–25 m above sea level), an isolated remnant of the vast extension of heathlands that once occupied northern Belgium, is covered by a mosaic of heathlands, moors, fens and woodlands. Although locally renowned for its diversity of heathland vegetation types, the landscape is uniform with inappreciable variation in altitude (range: 18–25 m), weather conditions and geology. The domain is entirely surrounded by agricultural land and residential areas that are totally unfavourable for adders, such that any permanent emigration out of our study area is likely to result in mortality. Immigration from other areas is non-existent, as the nearest adder sites are located at 18 km SSE (a small imperilled population), 110 km E, 115 km NE and 130 km S. Hence, the (meta)population in its entirety is effectively isolated.

Adders are found over the entire military domain, but we concentrated our searches to 11 study plots (1–8 ha each; total search area: 46.5 ha) that were chosen for high snake encounter rates, ease of access and no direct impact of military operations. To obtain a broad sample of the global (meta)population, study plots encompassed the variety of vegetation types present and their locations were dispersed over the area (mutual straight-line distances between the geographic centroids of the plots range between 280 m and 6,800 m, mean = 2,685 m, SE = 197 m).

Search areas are typical hibernation or “winter” habitats (Prestt, 1971) and covered by a dense (percent groundcover typically >95%) vegetation dominated by dwarf shrubs of common heather (*Calluna vulgaris*), cross-leaved heath (*Erica tetralix*) and bog-myrtle (*Myrica gale*), tussocks of purple moor grass (*Molinia caerulea*), patches of mosses and some localised thin groups of birch (*Betula pubescens*) and pine (*Pinus sylvestris*). From 2011 onwards we also searched in four feeding or so-called “summer” habitats (Prestt, 1971), mainly consisting of rough abandoned farmland, that were located 290–460 m distant from the corresponding hibernation grounds.

We visited each site several times per year and throughout the adders’ active season (late February to late October). For every visit to a sample locality we recorded date, start and end time, and number of trained field assistants participating. This enabled calculation of the number of person-hours per year, our index of annual searching effort.

Snakes were located by sight while walking slowly and erratically through the terrain, captured by hand and released immediately after handling. A digital photograph of the upper side of the head allows individual identification of adders, on the basis of the number, shape and arrangement of the head scales (Bauwens, Claus & Mergeay, 2018). At every encounter we also recorded date, time, exact location (GPS coordinates), sex, snout-vent (SVL; to nearest 5 mm), body mass (to nearest 1 g), breeding status of females (see below) and indications of recent food intake (midbody swelling, presence of touchable prey remnants, voiding of solid faeces).

The military commander of the “Groot Schietveld” and the “Agentschap voor Natuur en Bos (ANB)” (ANB/BL-FF/16-00002) gave permits to access the area and to study adders. All applicable institutional and national guidelines for the care and use of animals were followed.

### Ageing criteria

Aging assessments of individual snakes were based on their previous capture history and/or on SVL-criteria inferred from the growth trajectories of adders that were initially caught in their first, second or third active season (which can be easily recognized as such in the field; detailed description in Bauwens & Claus (2018). These data and criteria allowed us to distinguish between females that were in their third, fourth and fifth (or later) activity year. In the latter category more detailed age assignments were impossible due to high individual variation in growth rates, except for individuals first caught at a younger age. We note that snakes attain the actual age of *x* years during the second half of August (when the vast majority of births occur at our study area) of their *x* + 1th active year.

### Breeding status, body condition and reproductive output

Yearly reproductive status of individual adult females (reproductive/breeding versus non-reproductive/non-breeding) was determined by palpation of the abdomen to detect oviductal eggs or developing embryos and/or by signs of postparturient body condition (i.e., presence of extensive skin folds along the posterior one-third of the body). To avoid the mistaken assignment of non-breeding in a given year, this status was given only to females that did not show signs of pregnancy during June–August or had no indications of recent parturition in September–October.

We used a two-step process to calculate an index of body condition (BCI). First, we obtained the baseline relation between logMass and logSVL for female adders, including immature, non-reproductive and reproductive females, but excluding measurements taken after recent food intake or recent parturition (logMass (g) = −2.678 + 2.732 logSVL (cm), *R*^2^ = 0.968, *n* = 2297). Next, we estimated BCI as the difference between the observed mass and the mass predicted by the baseline relation, a procedure that is analogous to the calculation of regression residuals (Schulte-Hostedde et al., 2005). To avoid fluctuations of the BCI due to ingestion of food, we excluded estimates of BCI when signs of recent feeding were clearly visible.

Limitations of available time and lack of laboratory space, inherent to a citizen science project, impeded us to temporarily remove pregnant females from the field and house them in captivity until they gave birth. Therefore, we have no information on female mass (loss) at parturition, litter mass, the number of (viable) young born and their size at birth.

The body condition measured at the onset of the active season (March–half May) for vitellogenetic female adders (i.e., without oviductal eggs) provides a good indicator of the level of fat stores (Naulleau & Bonnet, 1996; Aubret et al., 2002). We examined whether SVL and BCI in spring influenced breeding status, as assessed at subsequent recaptures, with logistic regression. We also employed measurements of SVL and BCI during late summer (September–October) and related them to the reproductive status in the preceding summer. Because we lack measurements at parturition, our estimates for breeding females may be influenced by unnoticed feeding events or mass losses that occurred after giving birth.

We calculated BCI, and its relation to SVL, in early spring and late summer for all breeding and non-breeding females. Costs of reproduction should be reflected in how these population-level indices change over the active season in breeding versus non-breeding females. More direct estimates of annual changes in SVL and BCI were available for a restricted sample of individual females that were first caught and measured during spring (i.e., before ovulation) and again in late summer (i.e., post-parturient or end of active season) in the same year.

### Modelling of capture, transition and survival probabilities

We used mark–recapture data for adult females to estimate probabilities of capture, of transition between reproductive states and of survival. We collapsed daily capture data into yearly intervals, such that the encounter history file included for each individual snake a single entry (B/N/0 ; Breeding / Non-breeding / not captured) per year of study (2000–2017).

We used a Multistate capture-recapture model to estimate capture and annual survival probabilities associated with the current breeding state, and transition probabilities between breeding and non-breeding states conditional on survival. The initial model included state-dependent and temporal variation in capture, transition and survival probabilities. We first tested for goodness-of-fit of the data to the general time-dependent model in program U-CARE 2.3.4 (Choquet et al., 2009). Results suggested a good fit of the encounter data to the general time-dependent multistate model (Test 3G: Chi^2^ = 73.5, *df* = 98, *P* = 0.97; Test M: Chi^2^ = 34.7, *df* = 28, *P* = 0.18; cumulative test: Chi^2^ = 108.1, *df* = 126, *P* = 0.87). We then continued by fitting constrained versions of the initial fully time-dependent model. We used an information theoretic approach to rank models according to the sample-size adjusted Akaike’s Information Criterion (AICc) Burnham & Anderson, 2002; Anderson, 2008). To find the most parsimonious representation of the data, we used a step-wise procedure (Lebreton et al., 1992; Burnham & Anderson, 2002). We started model selection by searching for the most parsimonious structure for capture rate, while keeping maximal dimensionality for transition and survival probability. This included modelling the time dependence of capture rate as a hypothesized function of annual searching effort. In a second step, the resulting most parsimonious structure for capture rate was kept to model transition probabilities between reproductive states, maintaining maximal dimensionality for survival rate. Finally, the most parsimonious structures for capture and transition probabilities were used to model annual survival rate. Although we separately determined the modelling structure for capture, transition and survival probabilities, parameter estimates were obtained simultaneously and were calculated after model averaging over the entire model set (Burnham & Anderson, 2002; Anderson, 2008). All models were fitted using maximum-likelihood methods implemented in program MARK (White & Burnham, 1999; Cooch & White, 2015) through the RMark interface package (Laake & Rexstad, 2015) in R v3.5.1 (R Core Team, 2018). All modelling specifications follow procedures outlined in Laake & Rexstad (2015).

## Results

### Initiation of maturity and of annual breeding

None of the females known to be in their third activity year (*n* = 80) was reproductive. A small proportion of females in their fourth activity year (16% of *n* = 197), and a majority of those that were in at least their fifth year (73% of *n* = 1325) engaged in reproduction during a given year. Hence, most females start reproducing in their fifth or later activity year and thus produce their first litter upon attaining the actual age of 4 years or older.

Data from adult females that were in at least their fourth active season showed that the probability of reproducing in a given year increases with SVL attained during spring (March–April, i.e., before ovulation) (logistic regression, Chi^2^ = 17.4, 1 *df*, *P* < 0.01). The proportion of breeding females was zero at SVL <38 cm (0% of *n* = 19), increased to 75% (*n* = 105) in the size class SVL = 38–43 cm and to 88% (*n* = 263) in females with SVL ≥ 44 cm. Thus, we consider a SVL of 38 cm as the minimum size at maturity in females in our population.

**Figure 1:**
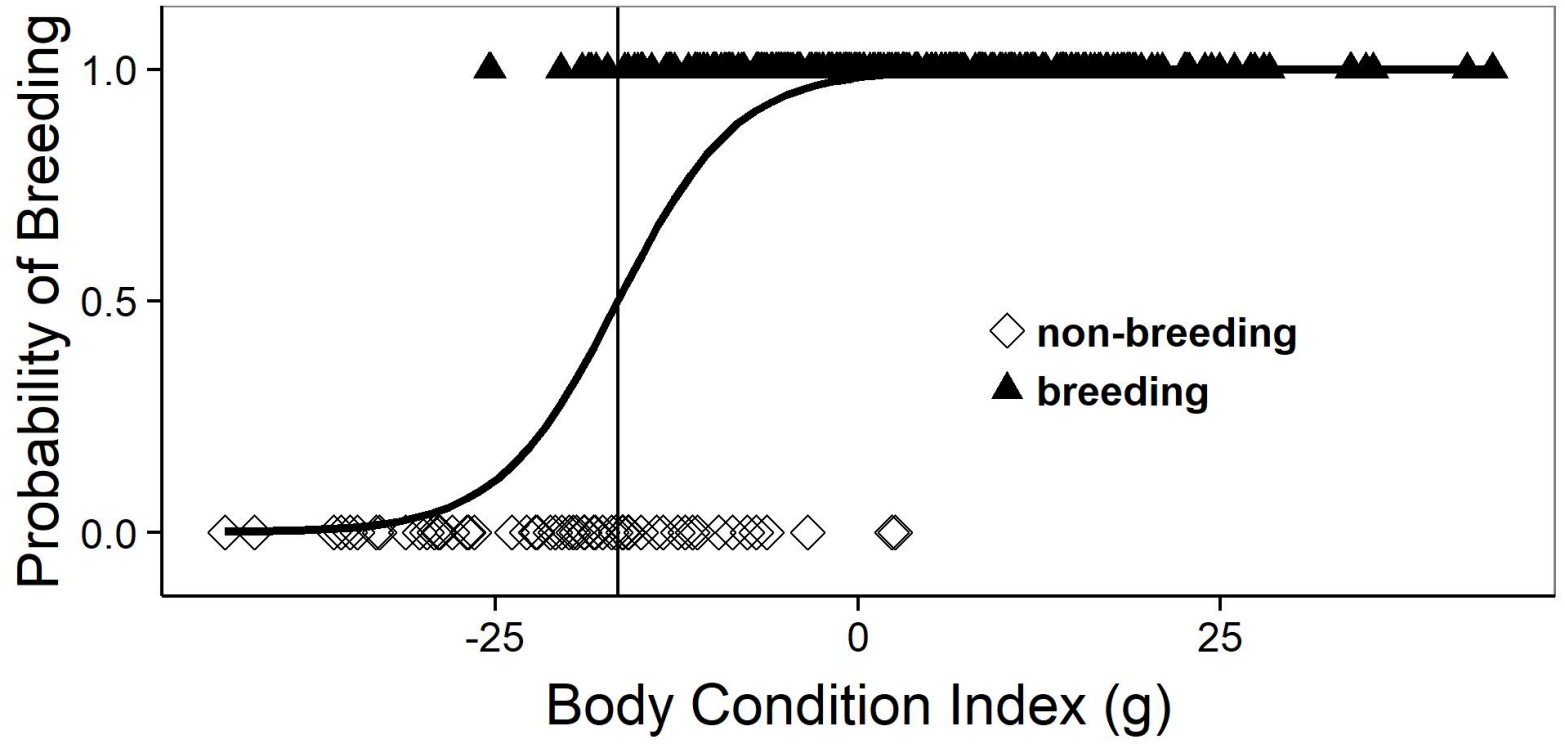
Logistic regression equation relating the annual probability of breeding to the body condition index during spring (March–half May; i.e., before ovulation). Each symbol depicts an individual female that was known to be breeding (triangles) or non-breeding (diamonds), as assessed at subsequent recaptures. The vertical line corresponds to the body condition index at which the breeding probability is 50%.

In the mature females (age ≥ 4th activity year and SVL ≥ 38 cm) captured during spring (March–half May, i.e., before ovulation) breeding probability increased sharply with body condition (logistic regression, Chi^2^ = 76.6, 1 *df*, *P* < 0.001). Thus, there seems to be a clear-cut body condition threshold for participating in reproduction (Fig. 1).

### Body condition and growth of breeders and non-breeders

**Figure 2:**
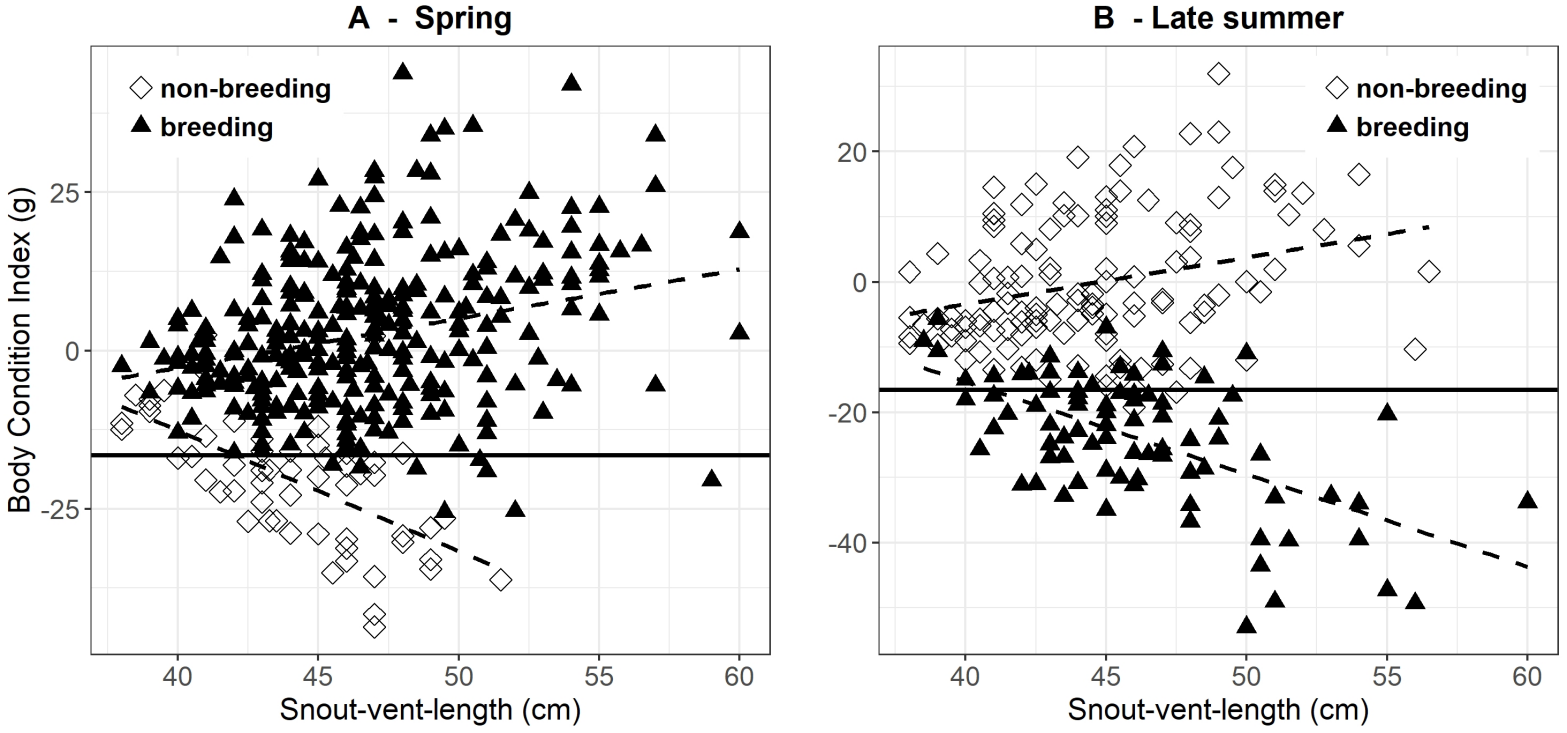
Body condition versus snout-vent length in breeding and non-breeding females during. Body condition versus snout-vent length in breeding (filled triangles) and non-breeding (open diamond) females during (A) spring (March–half May) and (B) late summer (September–October). The solid horizontal line corresponds to the body condition index in spring (i.e., before ovulation) at which the breeding probability is 50% (as determined by logistic regression; see Fig. 1). The dashed lines sketch the linear relations between body condition and SVL.

During spring, mean BCI was higher in breeding (2.4 g ± 0.7 SE, *n* = 310) than in non-breeding females (−20.5 g ± 1.3 SE, *n* = 58). Most females in the latter group had reproduced in the preceding year and had clearly not yet recuperated their BCI. There was a weak positive correlation between BCI and SVL in females in their breeding years (*r* = 0.277, *P* < 0.001), whereas this correlation was clearly negative in the non-breeding females (*r* =  − 0.616, *P* < 0.001) (Fig. 2). These patterns were reversed in late summer, i.e., after the breeding females gave birth (Fig. 2). Females that had reproduced had on average a much lower BCI (−23.8 g ± 1.1 SE, *n* = 86) than the non-reproductive females (−0.3 g  ± 0.9 SE, *n* = 130). Also, BCI was strongly negatively correlated with SVL in the breeding females (*r* =  − 0.589, *P* < 0.001), but moderately positive in the non-reproductive females (*r* = 0.281, *P* = 0.001). Hence, the difference in BCI between breeding and non-breeding females during late summer tended to be greatest in the larger females (Fig. 2).

We obtained estimates of changes in SVL, body mass and BCI in individual females that were measured during both spring (i.e., before ovulation) and late summer (i.e., post-parturient or at end of active season) within the same activity year (breeding: *n* = 34, non-breeding: *n* = 9). Non-breeding females grew on average 2.0 cm (± 0.6 SE), whereas breeding females did not increase in SVL (−0.1 cm ± 0.1 SE). Non-breeding females gained on average 29.0 g ± 6.5 SE or 69% over their initial body mass. Reproductive females lost on average 25.5 g  ± 2.6 SE, corresponding to a 32% decrease in body mass. Mass change of reproductive females was negatively correlated with their SVL (*r* =  − 0.657, *P* < 0.001) and mass (*r* =  − 0.744, *P* < 0.001) during spring.

### Capture, transition and survival probabilities

We built capture–recapture histories for 908 adult females and assessed their annual reproductive status for a total of 1,296 female-years (913 breeding and 383 non-breeding annual events). A majority of the females (=642 or 71%) were captured and their breeding status assessed during only one year; in the remaining females it was assessed during 2–7 annual occasions, spanning an interval of 2–11 activity years. Females that were captured in 2 or more consecutive years most often switched between breeding and non-breeding states (83% of *n* = 198). We observed two consecutive breeding years in 21 females (11%), two consecutive non-breeding years in 11 females (6%) and a single female reproduced during three years in a row.

**Figure 3:**
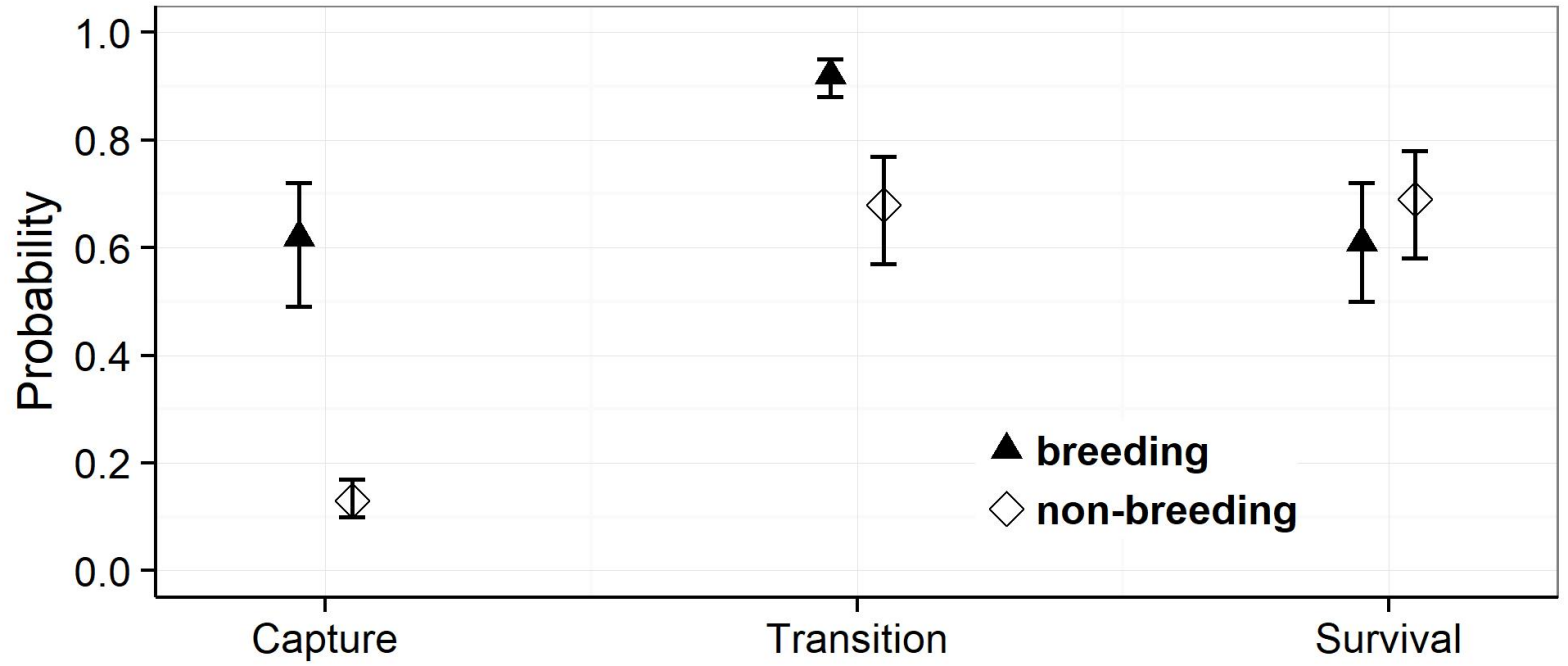
Estimates of the mean capture, transition and survival probabilities in breeding and non-breeding females. Mean estimates (symbols) as obtained by weighted averaging over the model set listed in Table 1, part (c). The vertical lines show the 95% confidence intervals.

**Figure 4:**
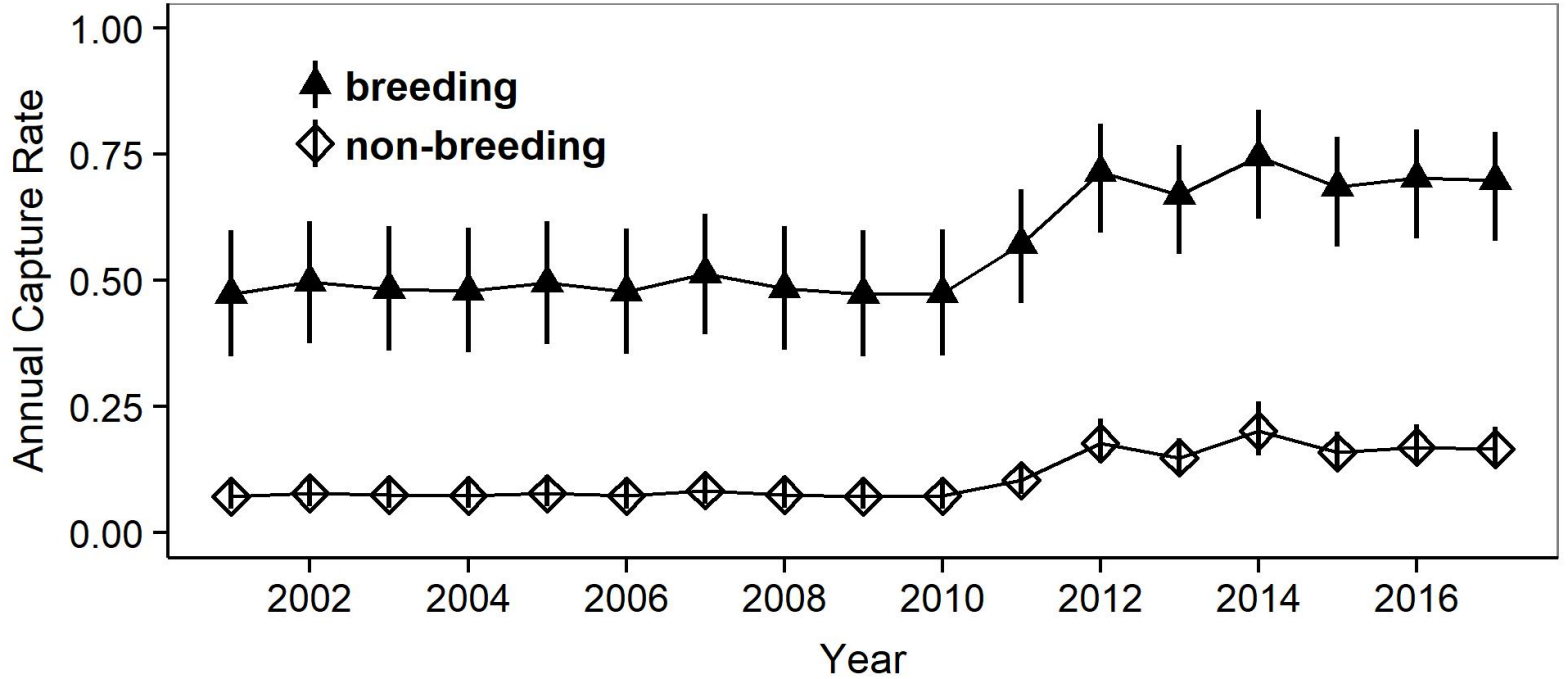
Estimates of mean annual capture probabilities in breeding and non-breeding females. Mean estimates as calculated by weighted averaging over the entire model set listed in Table 1, part (c). The vertical lines show the 95% confidence intervals. The higher capture rates after 2010 reflect a concomitant increase in capture effort (mean annual effort during 2000–2010 = 112.7 h, during 2011–2017 = 455.2 h).

Model selection procedures indicated that the most parsimonious model for capture probabilities included breeding status and annual capture effort as the main factors (Table 1). Overall, adult females were much more likely to be captured during their breeding years (*p* = 0.62, 0.49–0.72 (95% confidence limits)) than during non-reproductive years (*p* = 0.13, 0.10 –0.17) (odds ratio: 10.6; Fig. 3). Capture probabilities in both groups of females were considerably higher during the last 7 years of the study (2011–2017) than during the preceding years (2000–2010), concomitant with a parallel increase in capture effort (Fig. 4).

**Table 1:** Results of multi-state capture-recapture models for capture, transition and survival probabilities of adult female adders as a function of breeding status (state), time (year) and capture effort (Effort). Shown are the model name (number), model structure for Capture, Transition and Survival probabilities (the “*” sign denotes an interaction effect and the “+” sign an additive effect between two variables), the number of estimated parameters (K), Akaike’s information criterion corrected for small sample size (AIC_c_), the difference in AIC_c_ between each model and the most parsimonious model (Δ AIC_c_), and the ‘Akaike weights’ (weight) that assess the support that a given model has from the data, compared to the other models in the set. Three successive steps of model selection were employed (see text). The most parsimonious models in each step are shown in bold.

number	Capture	Transition	Survival	K	AIC_c_	Δ AIC_c_	weight
a) Modelling of capture probabilities							
**3**	**state + Effort**	state * year	state * year	**71**	**2517,3**	**0,0**	**0,736**
**4**	**state**	state * year	state * year	**70**	**2519,3**	**2,1**	**0,264**
5	state + year	state * year	state * year	86	2535,5	18,2	0,000
6	state * year	state * year	state * year	102	2560,8	43,5	0,000
2	Effort	state * year	state * year	70	2609,7	92,4	0,000
1	constant	state * year	state * year	69	2615,1	97,8	0,000
7	year	state * year	state * year	85	2618,7	101,4	0,000
							
b) Modelling of transition probabilities							
**9**	**state + Effort**	**state**	state * year	**39**	**2496,0**	**0,0**	**0,925**
13	state	state	state * year	38	2501,0	5,1	0,073
10	state + Effort	state + year	state * year	55	2510,0	14,1	0,001
8	state + Effort	constant	state * year	38	2510,8	14,8	0,001
14	state	state + year	state * year	54	2511,5	15,6	0,000
11	state + Effort	year	state * year	54	2516,1	20,1	0,000
3	state + Effort	state * year	state * year	71	2517,3	21,3	0,000
12	state	constant	state * year	37	2518,1	22,2	0,000
4	state	state * year	state * year	70	2519,3	23,4	0,000
15	state	year	state * year	53	2523,5	27,5	0,000
							
c) Modelling of survival probabilities							
**18**	**state + Effort**	**state**	**state**	**7**	**2468,6**	**0,0**	**0,574**
**19**	**state + Effort**	**state**	**constant**	**6**	**2469,3**	**0,7**	**0,400**
16	state + Effort	state	state + year	23	2475,9	7,3	0,015
17	state + Effort	state	year	22	2476,5	8,0	0,011
20	state	state	state + year	22	2483,1	14,5	0,000
21	state	state	year	21	2485,5	16,9	0,000
22	state	state	state	6	2487,1	18,5	0,000
23	state	state	constant	5	2488,3	19,7	0,000
9	state + Effort	state	state * year	39	2496,0	27,4	0,000
13	state	state	state * year	38	2501,0	32,4	0,000

The most parsimonious model for transition probability confirms that females alternate between breeding and non-breeding states (Table 1). The high probability of changing from breeding to non-breeding status (Psi = 0.92, 0.88–0.95; Fig. 3) shows that most breeding years are followed by a non-reproductive episode. The inverse probability (1 − Psi = 0.08) indicates that breeding in successive years occurs in ca 8% of the reproductive cycles, a number that agrees well with that obtained by direct observations (11%). The transition probability from non-breeding to breeding (Psi = 0.68, 0.57–0.77; Fig. 3) implies that about two thirds of non-breeding years are followed by a reproductive episode. The inverse probability (1 − Psi = 0.32) estimates that after about one third of the non-breeding years, females forego reproduction for another year and thus reproduce on a triennial schedule. This figure is much higher than that obtained through direct observations (6%).

For the probabilities of survival approximately equal support was obtained by the model that included breeding status as a factor and the model that assumed constant survival rates (Table 1). Parameter estimates attest to a slightly higher annual survival rate during non-breeding years (breeding: Phi = 0.61, 0.50–0.72: non-breeding: Phi = 0.69, 0.58–0.78) (odds ratio: 1.4), but confidence intervals overlap widely (Fig. 3). We note that our data provided very low support for models incorporating an additive or interactive effect of year on capture, transition and survival probabilities. Thus, that transition probabilities, and hence frequencies of reproductive states, and annual survival rates did not vary significantly among years during the course of this study.

### Lifetime reproductive frequency

**Figure 5:**
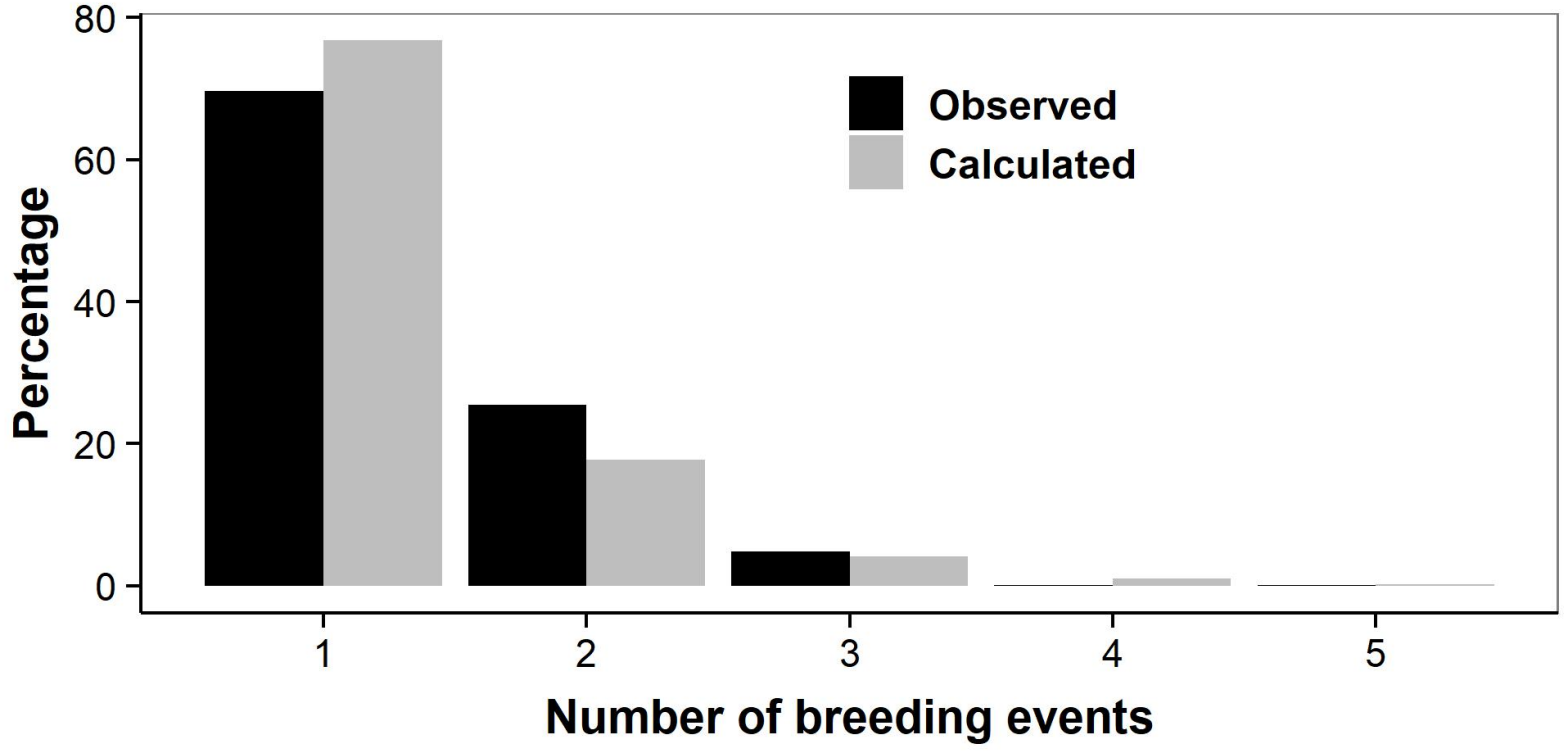
Number of lifetime breeding years in individual female adders. Observed: actual number of breeding years counted in 483 individuals; to avoid bias, females that were first captured during the last four study years (2014–2017) were not included (altering the arbitrary cut-off year did not affect the results). Calculated: number of breeding years based on the likelihood-based estimates of survival and transition probabilities.

We counted the number of breeding years observed in successive annual samples of individual females that were sexually mature. We removed the females that were first captured during the last four study years (2014–2017), as they could most likely only be observed breeding once or twice, and would bias the result. A vast majority of the females was observed breeding only once (77% of *n* = 483; Fig. 5) or twice (18%). Only 5% of adult females lived long enough to reproduce three or more times, with an observed maximum of five reproductive events in a single female. Females thus produced on average only 1.30 litters in their lifetimes.

As we did not capture all females that were present (i.e., capture rates were <1), our counts might have underestimated the actual number of breeding events. We therefore also calculated lifetime breeding frequency based on the likelihood-based estimates of survival and transition probabilities, which account for variation in capture rates. The results confirmed our former calculations: 70% of the adult females reproduce only once (Fig. 5), the mean number reproductive efforts per lifetime being 1.35.

## Discussion

Our analyses of breeding and non-breeding individual adders succeeded in answering the main questions posed in the introduction. (1) Females mature upon attaining a minimum snout-vent-length, rather than a minimum age; (2) A threshold level of energy reserves is necessary to trigger the initiation of a reproductive cycle; (3) During breeding years, females lose body mass and stop growing; (4) We found no evidence for a difference in annual survival rate between breeding and non-breeding individuals; (5) The reproductive cycle was biennial or triennial but a majority of the females breed only once in their lifetime.

Our main conclusions were obtained by explicitly incorporating the variation in capture rate in our analyses. This is required to obtain accurate and robust likelihood-based estimates of survival and transition probabilities, and hence (lifetime) breeding frequency (Lebreton et al., 1992; Baron et al., 2013; Mazerolle, 2015). Although we are mainly interested in the latter variables, understanding the differences in detection rates can reveal important aspects of the study species’ behaviour and natural history (Lourdais et al., 2002).

### Variation in capture probabilities

Adult female adders were much more likely to be captured in breeding than in non-breeding years (odds ratio: 10.6). Comparable differences in capture rates associated with reproductive status have been reported for the closely related aspic viper (*V. aspis*) and meadow viper (*V. ursinii*) (Bonnet et al., 2002; Baron et al., 2013). Breeding adders typically concentrate in hibernation or “winter” areas, where they spend the winter and where most reproductive activities (mating, pregnancy, parturition) take place (Prestt, 1971; Völkl & Thiesmeier, 2002; Bauwens & Claus, 2019a). Gravid adders are very sedentary, showing extremely limited movements (Madsen & Shine, 1992) and are frequently seen at very specific microhabitats that facilitate thermoregulation (Bauwens & Claus, 2019a). They regulate their body temperature at higher levels and more precisely than non-reproductive females (Lourdais et al., 2013), presumably to enhance development of the embryos. By spending much time to overt thermoregulatory behaviours, such as basking or simply lying out during suboptimal weather conditions, they are more exposed to human snake-catchers, resulting in high encounter and capture rates (Bauwens & Claus, 2019a).

By contrast, during non-reproductive years females become almost invisible to snake-catchers, very much like the elusive immature adders (Bauwens & Claus, 2018). This is reflected in the low capture rates of the non-breeding females (on average p ≈ 0.1), indicating that we captured on a yearly basis only one out of ten individuals that were present. During their non-reproductive years, females spend most of the active season in the feeding or “summer” areas, where food is more abundant (Viitanen, 1967; Prestt, 1971; Luiselli et al., 1994; Völkl & Thiesmeier, 2002; Phelps, 2004). These are spread out over a large area and diverse habitat types that have extremely dense vegetation, hampering adder observations (Prestt, 1971), and were also less frequently visited by us. Thus, the low detection rates of the non-breeding females should mainly be attributed to their temporary migration out of the hibernation areas. In addition, non-breeding female adders have lowered thermal needs and spend more time under cover (Lorioux, Lisse & Lourdais, 2013).

An inevitable consequence of the low detection rates of non-breeding females is the difficulty to obtain repeated captures of individual females within the same year. Hence, the low sample size for variables requiring consecutive captures in spring and autumn (i.e., before ovulation and after parturition).

### Sexual maturity and initiation of reproduction

The attainment of sexual maturity in female adders depends more on body size than on age. The minimum SVL at maturity was 38 cm at our study site, and agrees well with the minimum SVL of 40 cm in a British population (Prestt, 1971). A minority of the females reached this size at the onset of their 4th activity year and gave birth to their first litter later that year upon attaining the actual age of 3 years. However, most females reproduced for the first time upon attaining the actual age of 4 years or more. In mature females, breeding in a given year depends to a large extent on their body condition at the onset of the active season. This indicates that a threshold level of energy reserves is necessary to start a reproductive cycle, similarly as in the aspic and meadow viper (Naulleau & Bonnet, 1996; Baron et al., 2013).

Breeding imposes considerable investment of energy in the formation of yolk and the metabolic costs of pregnancy (Ladyman et al., 2003; Van Dyke & Beaupre, 2011). In addition, females of many Old-world viperids reduce or even completely cease feeding during pregnancy (Prestt, 1971; Bea et al., 1992; Bonnet et al., 2001; Lourdais, Bonnet & Doughty, 2002; Bauwens & Claus, 2019b). Consequently, female adders in our population do not grow during their breeding years, lose on average ca. 25 g or about one-third of their initial body mass and are very emaciated after parturition. The decrease in body mass was negatively correlated with SVL and mass before ovulation. Hence, the initially largest and fattiest females lost the highest proportion of their original mass and were most emaciated after parturition (see also Madsen & Shine (1993)). These results suggest that females with the best condition in spring devoted proportionally more energy to the litter, or alternatively faced higher metabolic costs during pregnancy. As we could not obtain precise estimates of reproductive output (litter size and mass) and of maternal mass loss, we cannot discriminate between these two putative mechanisms. Neither can we explore whether there is a high fecundity-independent component to the energy cost of reproduction, as indicated by results of Madsen & Shine (1993). Anyhow, the largest females especially have a weakened general condition after giving birth and go into hibernation with reduced energy reserves.

At the onset of their non-breeding years, most females have not yet recovered from their preceding breeding investment: their body condition is on average low, and this is especially so in the largest females, mirroring the situation at the end of a breeding year. They will reside most of the non-breeding year in the feeding habitats and forage intensely to rebuild their fat reserves. Females that after one non-breeding year accumulate energy reserves that surpass the threshold level for the induction of vitellogenesis, will start a new reproductive year. Females that fail to amass the necessary amount of lipid stores, will postpone reproduction for another year (or longer). The rate at which a female adder can replenish her energy stores after a breeding year will therefore strongly affect whether and when she engages in subsequent reproductive bouts, and hence her lifetime reproductive frequency.

### Mortality during breeding and non-breeding years

Several physiological and behavioural modifications associated with reproduction are expected to result in increased mortality rates. First, gravid females devote more time to thermoregulation and thereby supposedly expose themselves for longer periods to predation (Andrén, 1985; Aubret et al., 2002; Bonnet et al., 2002; Baron et al., 2013; Lorioux, Lisse & Lourdais, 2013; Lourdais et al., 2013). Second, breeding females are physically burdened by the weight and volume of the litter (Nilson, 1981; Lorioux, Lisse & Lourdais, 2013), reducing their abilities to escape predation by swift fleeing. In addition, post-parturient females are extremely emaciated and may suffer high mortality, either because they die of starvation or because they are taken by predators while searching for food (Madsen & Shine, 1993; Bonnet et al., 2002). Increased mortality during reproductive years has been documented in the adder (Madsen & Shine, 1993) and the aspic viper (Bonnet et al., 2002). By contrast, our present analyses and those of Baron et al. (2013) for the meadow viper, did not reveal a substantial difference in annual survival rates between females in breeding and non-breeding state.

The different results obtained by these studies may, to some extent, be a consequence of analytical methods used. Our analyses and those of Baron et al. (2013) explicitly account for the differences in capture rate associated with breeding state, a necessary procedure to obtain accurate estimates of survival probabilities (Lebreton et al., 1992; Mazerolle, 2015). By contrast, Madsen & Shine (1993) and Bonnet et al. (2002) estimated mortality rates by the proportion of individuals that were recaptured, neglecting the potential confounding effects of differences in capture probabilities. As these differences are large (Bonnet & Naulleau, 1996; Baron et al., 2013; Figs. 3 &  4) mortality estimates that do not account for capture rates may well be imprecise and biased.

Given our long-term (18 years) study, large samples and usage of adequate analytical methods, we are confident that we correctly assessed that annual survival rates of breeding and non-breeding females are not different. Hence, activities directly related to reproduction (increased basking, physical burden) do not seem to negatively affect the females’ survival abilities.

A more detailed analysis of the within-year (i.e., seasonal) variation in survival rates showed differences in the temporal patterns during breeding and non-breeding years (Bauwens & Claus, 2019a). In reproductive females mortality was virtually absent during spring and summer, a result that concords with that of Madsen & Shine (1993). Mortality was higher after giving birth and throughout the hibernation period, although the increase was not as dramatic as observed in the population studied by Madsen & Shine (1993). In our population the mortality rates were clearly highest during the spring period of the ensuing non-breeding years (Bauwens & Claus, 2019a). After giving birth, breeding females have a weakened body condition and it takes several active months to recover from this status. Due to their weakened general condition, post-parturient females run the risk of dying from starvation either before, during or after hibernation. In addition, they may experience increased susceptibility to predation while foraging and during migrations to the feeding grounds (Madsen & Shine, 1993; Bonnet et al., 2002). The elevated mortality during spring of the non-reproductive years should thus be considered as an indirect and delayed survival cost induced by the high investment in previous vitellogenesis and the reduced feeding during pregnancy (Bauwens & Claus, 2019a). The total survival cost of reproduction for a female adder is thus spread over an extended period, not only encompassing mortality directly related to breeding activities, but also mortality associated with recovering from the weakened post-parturient body condition during non-breeding years (Bonnet et al., 1999; Sperry & Weatherhead, 2009).

### Breeding frequency

The estimates of transition probabilities between breeding states indicate that the relative frequency of the length of the interval between consecutive reproductive bouts in our study population was one (8%), two (63%) or three (29%) years. Female adders thus reproduce with a predominantly biennial or triennial frequency, matching results obtained in most other studies of this species (Prestt, 1971; Nilson, 1981; Madsen & Shine, 1993; Völkl & Thiesmeier, 2002; Nilson, Andrén & Völkl, 2005). Yet, a rather high incidence of breeding in successive years was indicated by both estimates of transition probabilities (8%) and direct observations (11% of observed 2-year cycles). Annual breeding was also detected in a mountain population of the adder (Strugariu, Gherghel & Zamfirescu, 2014). The probability of changing status after a non-breeding year showed that about two-thirds of non-breeding years are followed by a reproductive episode, resulting in a biennial reproductive frequency. In the remaining one third (32%) of cases, females forego reproduction for another year and thus reproduce on a triennial schedule. This frequency is much higher than the estimate (6%) obtained through direct observations of the reproductive status of individual females in successive years. This notable difference is explained by considering the difference in annual capture rates in relation to reproductive status. Due to the low capture probabilities of non-breeding females, we might indeed expect that instances of consecutive non-breeding years go undetected quite often and much more frequently than occurrences of successive reproduction. Hence, failure to consider variation in detectability, which is essentially equal to assuming that capture probability is perfect or constant, leads to erroneous estimates of breeding frequency (Bonnet & Naulleau, 1996).

One of our main results is that females produced on average only ca. 1.3 litters per reproductive lifetime. Most individuals (ca. 70%) that attained sexual maturity reproduced only once, while a minority of the females (ca. 5%) were observed to breed 3–5 times. These findings, obtained in our large population, parallel those of Madsen & Shine (1992) for a small adder population.

A short reproductive lifespan and a strong tendency toward a single lifetime reproductive bout (i.e., semelparity) is also observed in other viperids (Bonnet et al., 2002; Baron et al., 2013; Brown, 2016). These species deviate from a pattern of delayed maturity combined with multiple reproductive cycles that has been documented in other long-lived squamate reptiles (Parker & Plummer, 1987; Dunham, Miles & Reznick, 1988). Delayed maturation is only likely to evolve when juvenile and immature survival rates are very high (Madsen & Shine, 1992). This suggestion is supported by detailed studies showing that annual survival rates are, at most, only slightly lower in immature than in adult timber rattlesnakes, meadow vipers and adders (Brown, Kéry & Hines, 2007; Baron et al., 2010; Bauwens & Claus, 2018).

The observation that only a small number of females manage to reproduce multiple times in their lifespan, implies that they may leave a disproportionally high number of offspring (Brown, 2016). This may tend to reduce the effective population size and hence to potentially increase the loss of genetic variation (Ridley, 2004). The observed individual variation in lifetime reproductive frequency is presumably related to differences among females in their abilities to survive and to gather resources to fuel reproduction. Differences in foraging success are especially relevant when the consumption of single prey items has a significant impact on the total energy budget. Adders are ambush foragers (Glaudas et al., 2019), a strategy that precludes a high feeding rate and hence rapid energy recovery (Greene, 1983). They typically feed on small mammals (mice, voles, shrew; Völkl & Thiesmeier (2002) and the mass of such a single prey item amounts to ca. 20–35% of the body mass of an adult female adder. The capture of a few prey items more or less may thus have a large effect on the amount of body reserves and can thereby influence (lifetime) reproductive frequency.

## Conclusions

We studied various aspects of the reproductive biology of adders (*Vipera berus*), using extensive data collected during an 18-year (2000–2017) mark–recapture study in a large population of adders. The attainment of sexual maturity in female adders depends more on body size than on age. The minimum SVL at maturity was 38 cm and a minority (16%) of the females reached this size and gave birth to their first litter at the actual age of 3 years. However, most females delay their first reproductive event to the age of 4 years or more. The probability that a mature female will reproduce in a given year depends to a large extent on her body condition at the onset of the active season, indicating that a threshold level of energy reserves is necessary to start a reproductive cycle. Breeding females invest their energy (fat) reserves in the formation of yolk, reduce or even completely cease feeding during pregnancy, stop growing, lose about one third of their initial body mass and are very emaciated after parturition. Their abilities to replenish energy stores after giving birth to their young will determine whether and when they engage in subsequent reproductive bouts. The length of the interval between consecutive reproductive bouts was one (8%), two (63%) or three (29%) years; hence female adders reproduce with a predominantly biennial or triennial frequency. Because most individual females do not produce litters every year, breeding and non-breeding females are concurrently present in the population, providing an opportunity to synchronously estimate their survival rates. Multistate mark–recapture analytical models indicated much higher capture rates during the breeding years, but provided no evidence for a substantial effect of reproductive state on annual survival rates. Most individual females (ca. 70%) that attained sexual maturity reproduced only once, while a minority of the females (ca. 5%) were observed to breed 3–5 times. On average, females produced only ca. 1.3 litters during their reproductive lifetime. The strong tendency toward a single lifetime reproductive bout (i.e., semelparity) is also observed in other viperids and deviates from the pattern of delayed maturity combined with multiple reproductive cycles that has been documented in other long-lived squamate reptiles.

## Additional Information and Declarations

### Field Study Permissions

The following information was supplied relating to field study approvals (i.e., approving body and any reference numbers): The military commander of the ‘Groot Schietveld’ and The Flemish Government, Agency of Nature and Forest provided full approval and permits for this research (ANB/BL-FF/16-00002).

### Data Availability

The following information was supplied regarding data availability: Dirk Bauwens, & Katja Claus. (2018). Intermittent reproduction, mortality patterns and lifetime breeding frequency of females in a population of the adder (Vipera berus) [Data set]. Zenodo. http://doi.org/10.5281/zenodo.2527132.
